# Comparison of CNN-Learned vs. Handcrafted Features for Detection of Parkinson's Disease Dysgraphia in a Multilingual Dataset

**DOI:** 10.3389/fninf.2022.877139

**Published:** 2022-05-30

**Authors:** Zoltan Galaz, Peter Drotar, Jiri Mekyska, Matej Gazda, Jan Mucha, Vojtech Zvoncak, Zdenek Smekal, Marcos Faundez-Zanuy, Reinel Castrillon, Juan Rafael Orozco-Arroyave, Steven Rapcsak, Tamas Kincses, Lubos Brabenec, Irena Rektorova

**Affiliations:** ^1^Department of Telecommunications, Brno University of Technology, Brno, Czechia; ^2^Intelligent Information Systems Laboratory, Faculty of Electrical Engineering and Informatics, Technical University of Kosice, Košice, Slovakia; ^3^Escola Superior Politecnica, Tecnocampus, Mataró, Spain; ^4^Faculty of Engineering, Universidad de Antioquia—UdeA, Medellín, Colombia; ^5^Faculty of Engineering, Universidad Católica de Oriente, Rionegro, Colombia; ^6^Pattern Recognition Lab, Friedrich-Alexander-Universität, Erlangen, Germany; ^7^Department of Neurology, College of Medicine, University of Arizona, Tucson, AZ, United States; ^8^Department of Neurology, University of Szeged, Szeged, Hungary; ^9^Applied Neuroscience Research Group, Central European Institute of Technology—CEITEC, Masaryk University, Brno, Czechia; ^10^First Department of Neurology, Faculty of Medicine and St. Anne's University Hospital, Masaryk University, Brno, Czechia

**Keywords:** machine learning, deep learning, feature extraction, Parkinson's disease dysgraphia, handwriting analysis

## Abstract

Parkinson's disease dysgraphia (PDYS), one of the earliest signs of Parkinson's disease (PD), has been researched as a promising biomarker of PD and as the target of a noninvasive and inexpensive approach to monitoring the progress of the disease. However, although several approaches to supportive PDYS diagnosis have been proposed (mainly based on handcrafted features (HF) extracted from online handwriting or the utilization of deep neural networks), it remains unclear which approach provides the highest discrimination power and how these approaches can be transferred between different datasets and languages. This study aims to compare classification performance based on two types of features: features automatically extracted by a pretrained convolutional neural network (CNN) and HF designed by human experts. Both approaches are evaluated on a multilingual dataset collected from 143 PD patients and 151 healthy controls in the Czech Republic, United States, Colombia, and Hungary. The subjects performed the spiral drawing task (SDT; a language-independent task) and the sentence writing task (SWT; a language-dependent task). Models based on logistic regression and gradient boosting were trained in several scenarios, specifically single language (SL), leave one language out (LOLO), and all languages combined (ALC). We found that the HF slightly outperformed the CNN-extracted features in all considered evaluation scenarios for the SWT. In detail, the following balanced accuracy (BACC) scores were achieved: SL—0.65 (HF), 0.58 (CNN); LOLO—0.65 (HF), 0.57 (CNN); and ALC—0.69 (HF), 0.66 (CNN). However, in the case of the SDT, features extracted by a CNN provided competitive results: SL—0.66 (HF), 0.62 (CNN); LOLO—0.56 (HF), 0.54 (CNN); and ALC—0.60 (HF), 0.60 (CNN). In summary, regarding the SWT, the HF outperformed the CNN-extracted features over 6% (mean BACC of 0.66 for HF, and 0.60 for CNN). In the case of the SDT, both feature sets provided almost identical classification performance (mean BACC of 0.60 for HF, and 0.58 for CNN).

## 1. Introduction

Parkinson's disease (PD) is a chronic idiopathic disorder characterized by the progressive loss/degeneration of dopaminergic neurons in the *substancia nigra pars compacta* (Hornykiewicz, 1998; Dickson, 2012) with the development of α-synuclein-containing Lewy bodies within the dopaminergic neurons (Forno, 1996). PD is the second most frequent neurodegenerative disorder, with the prevalence rate estimated to be ~2.0% for people aged over 65 years (Heinzel et al., 2019). To date, the gradual deficiency of dopaminergic neurons in the basal ganglia has been recognized as a major cause of parkinsonian symptoms (Brodal, 2003). In addition to a large variety of other motor symptoms, such as tremor at rest (Hughes et al., 1993), progressive bradykinesia (Berardelli et al., 2001), muscular rigidity (Hughes et al., 1993), postural instability (Horak et al., 2005), and hypokinetic dysarthria (Brabenec et al., 2017), one of the prominent motor symptoms of PD is so-called Parkinson's disease dysgraphia (PDYS) (Letanneux et al., 2014; Pinto and Velay, 2015; Thomas et al., 2017).

PDYS is a term describing a spectrum of neuromuscular difficulties, including motor-memory dysfunction (problems combining memory input with motor output), graphomotor production deficits (poor muscle coordination), motor feedback difficulties (over-activation of certain muscles and joints during handwriting as well as problems tracking the location of the pen's tip) and others. These cause a variety of handwriting difficulties (HD) manifesting as dysfluent, shaky, slow, and less readable handwriting; a progressive decrease in letter amplitude or width, namely, micrographia (McLennan et al., 1972; Rosenblum et al., 2013; Letanneux et al., 2014); etc. Hence, PDYS has serious consequences that significantly affect a person's everyday life, starting with slow and less legible handwriting and often progressing to lower self-esteem, poor emotional well-being, problematic communication, and social interaction, and many others. To introduce a timely and effective treatment to improve a patient's quality of life as much as possible, neurologists, and other experts could benefit from a remote, objective, fast, and low-cost decision support system. Such a system could employ artificial intelligence and provide information that might lie beyond human perception. It could enable specialists to combine their expertise with a large volume of data that are not available when utilizing a conventional in-clinic examination to identify and assess parkinsonian symptoms. Finally, such an approach could be implemented in decentralized clinical trials and could significantly suppress the Hawthorne effect (Morberg et al., 2018).

In general, the handwriting tasks that are traditionally employed in PDYS analysis can be classified into drawing, writing, and more complex tasks (Vessio, 2019). Usually, simple drawing or writing elements are performed repetitively and continuously as a single exercise. In the drawing task category, spirals, circles, meanders, and simple figures are frequently used for motor performance evaluation. These types of drawing tasks are effortless and well-tolerated and hence are suitable for studying motor control deficits in PD patients, especially for assessing tremor (San Luciano et al., 2016; Vessio, 2019). As PD patients commonly exhibit constructional apraxia (Garre-Olmo et al., 2017), their drawings may contain simplifications, lack of perspective, fewer angles, or spatial alterations. Letters, words, and sentences are commonly acquired during the examination process in the writing task category. As PD patients may produce slower and more irregular movements, mainly due to rigidity and bradykinesia, the results of repetitive writing tasks usually emerge in a more segmented fashion (Pullman, 1998; Drotar et al., 2016). Sentence writing requires a high degree of simultaneous processing, including motor planning; therefore, it is suitable for detecting micrographia (Bidet-Ildei et al., 2011), which is the most commonly observed handwriting abnormality in PD patients. Finally, more complicated handwriting tasks, such as the Clock Drawing Test (Agrell and Dehlin, 1998), may be used as well as part of a more complex examination involving cognitive and functional issues.

Currently, the most promising approach for the robust, objective, and computerized assessment of PDYS utilizes various signals describing the process/product of handwriting acquired by a digitizing tablet (Drotar et al., 2014, 2015). Such signals represent the movement of a digitizing stylus (pen) along both the horizontal and vertical axes, the pressure exerted on the surface of a digitizer, and the tilt and azimuth angles, acquired with respect to a specific series of timestamps to form a collection of time series describing the process of handwriting from beginning to end (referred to as online handwriting). In addition, modern digitizers have the ability to record not only the movement of a pen on the surface of the digitizer but also the movement above the surface (in-air movement; Alonso-Martinez et al., 2017). As shown in a variety of research studies focusing on the identification and assessment of HD in patients suffering from PD, Alzheimer's disease (AD), essential tremor (Drotar et al., 2014, 2016; Alonso-Martinez et al., 2017; Impedovo et al., 2018), etc., online handwriting capture provides the ability to characterize the process of handwriting in terms of its kinematic, dynamic, and temporal features, which are not accessible from the final handwritten product when using the conventional pen and paper methodology (referred to as offline handwriting).

At present, the following handcrafted features are conventionally used to describe the product/process of handwriting/drawing (Rosenblum et al., 2013; Thomas et al., 2017; De Stefano et al., 2019): (a) spatial features—width, height, and length; (b) temporal features—duration; (c) kinematic features—velocity, acceleration, and jerk; (d) dynamic features—pressure, tilt, and azimuth; and (e) other features—number of interruptions (pen elevations), etc. These features are computed either for an entire product or on a per-stroke basis utilizing on-surface and in-air movements. In the case of per-stroke computation, the investigated signals are broken down into the separate strokes forming the final handwritten product. A crucial characteristic of these conventional features is their clinical interpretability, allowing them to be linked with the real physiological phenomena behind the studied pathologies, which is extremely important for the mass adoption of this methodology in real clinical use cases.

Despite the broad use and indisputable success of these conventional handcrafted features, our recent studies (Mucha et al., 2018a,b; Mucha et al., 2019) concerning the computerized identification and assessment of PD and developmental dysgraphia (DD) have illustrated the necessity of additional research into novel and more advanced parametrization techniques for handwriting that could enable more robust and complex characterization of HD. For this reason, various nonlinear handwriting features based on modulation spectra, fractional-order derivatives (FD) and the tunable-Q wavelet transform have been developed and evaluated (Galaz et al., 2020; Mucha et al., 2020).

Conventional and nonlinear handcrafted features have shown promising potential for the quantification of hidden patterns in deficient handwriting. However, the necessity of manual design and development is still a severe limitation. Recent advancements in artificial neural networks offer new possibilities for automated feature extraction. By utilizing transfer learning, pre-trained convolutional neural networks (CNNs) can be advantageously used to extract features and, as such, provide an alternative solution in place of tedious and time-consuming manual feature design. This approach has already been used not only for handwriting processing (Gil-Martin et al., 2019; Moetesum et al., 2019; Gazda et al., 2021) but also in several other domains (Hagerty et al., 2019; Minaee et al., 2020). Nevertheless, in the area of handwriting processing, one apparent limitation of CNN feature extraction is that it utilizes only image data, and as such, it is limited only to offline handwriting processing. However, there have recently been some promising attempts to employ recurrent neural networks for the classification of handwriting signals (Diaz et al., 2021).

As seen from the above discussion, various parametrization techniques for offline and online handwriting have been developed. However, a major limitation of the current state of affairs is that these techniques are treated separately most of the time. Studies comparing the robustness of conventional handcrafted features with that of features extracted automatically using a pre-trained CNN for the identification and assessment of PDYS are lacking. Moreover, multilingual studies analyzing datasets acquired from subjects of different nationalities are very rare.

The primary goal of this work is to compare two different approaches for the identification of PDYS from drawing and handwriting. The first approach is based on online handwriting utilizing a set of conventional handcrafted features (baseline), whereas the second approach relies on automated feature extraction from offline handwriting utilizing a pre-trained CNN. The primary aim of this comparison is to reveal whether a set of features that are automatically extracted with no prior domain knowledge could compete with a set of handcrafted features designed by domain experts. The secondary goal of this work is to explore the power of both feature sets for the identification of PDYS in a multilingual dataset. In this study, we consider two different handwriting tasks, namely, the Archimedean spiral drawing task and the sentence writing task. The reason behind this selection is to examine a drawing task, which is independent of language, and a writing task, which is dependent on language. We note that except for our own previous work (Mucha et al., 2019), in which the Spanish and Czech sentence tasks were investigated together, this is the only study to date to consider a large multilingual cohort of PD patients, who were enrolled in the Czech Republic, the United States, Colombia, and Hungary. Such cross-language and cross-cultural clinical studies are essential to generalize the methodology used for PDYS diagnosis and assessment; therefore, the findings of this study could lay a foundation for future research in this area.

## 2. Related Works on PD Classification From Handwriting

### 2.1. Online Handwriting

The most frequently used handcrafted features extracted from online handwriting can be divided into (a) conventional features (temporal, spatial, kinematic, and dynamic) and (b) advanced features (Vessio, 2019). Among conventional features, the following features have been utilized the most: (a) temporal—duration of writing, duration of strokes; (b) spatial—width, height, and length of a written product or of individual strokes; (c) kinematic—velocity, acceleration, jerk; and (d) dynamic—pressure, tilt, azimuth, etc. With respect to advanced features, various studies have explored designs based on entropy, the signal-to-noise ratio (SNR), empirical mode decomposition (EMD), cepstrum (Nolazco-Flores et al., 2021), sigma–lognormal models (O'Reilly and Plamondon, 2009), FD (Mucha et al., 2018b), etc.

To obtain a complete picture of the utilization of handcrafted features in PDYS diagnosis and assessment, we refer to comprehensive reviews published up through 2019 (Letanneux et al., 2014; Impedovo and Pirlo, 2018; De Stefano et al., 2019; Vessio, 2019). In the following discussion, we review a number of recent articles. Although the present work investigates conventional features only, the review below includes studies that have employed conventional features, advanced features, or both; the primary focus is the summarization of the latest works addressing the computerized assessment of HD in patients suffering from PD.

Impedovo et al. (2018) investigated whether a diagnosis of PD based on the quantitative analysis of online handwriting could be successful in early to mid stages of the disease. For this purpose, the PaHaW database was reduced to a subset of 65 subjects [36 healthy controls (HCs) and 29 PD patients] who fit the Hoehn and Yahr scale at scores from 1 to 2.5 (Goetz et al., 2004, 2008). Almost all of the extracted features were kinematic, whereas some of them utilized entropy. Significant discriminative power was achieved in the sentence task [accuracy (ACC) of 71.95% with a Gaussian naïve Bayes classifier], thus confirming the previously reported findings of Drotar et al. (2016) that the writing of a long sentence presents a higher cognitive demand such that the effect of PD can manifest itself in the aggravation of HD.

Intending to improve the computerized assessment of PD severity, Mucha et al. (2018b) deeply analyzed various advanced kinematic features based on FD. The newly designed features were compared to conventional ones for only those PaHaW subjects who completed all of the 9 tasks (Drotar et al., 2016) (69 subjects in total). The authors reported that the conventional in-air features outperformed the advanced ones in the differential analysis (ACC of 97.1% with an XGBoost classifier) as well as in the estimation of PD duration [estimation error rate (EER) of 23.6%], but in this specific case, the in-air parameters were combined with features extracted from the on-surface movement. On the other hand, the severity of PD in terms of the score on the Unified Parkinson's Disease Rating Scale, part V: Hoehn and Yahr scale (UPDRS V) was better estimated by the new FD-based metrics (EER of 12.5%), suggesting that fractional calculus can play a significant role in the assessment of PD.

In 2019, Rios-Urrego et al. (2019) analyzed the ability to use kinematic, geometric, spectral and nonlinear dynamic features to model HD and to discriminate between HCs and patients with PD. In that study, they enrolled 130 subjects from Colombia, who were asked to draw an Archimedean spiral and to write a short sentence. The results indicated an ACC of 83.3% [K-nearest neighbors (KNN) classifier] for the Archimedean spiral and ACC of 75% [support vector machine (SVM) classifier] in the case of the sentence writing task. The absence of nonlinear features in the trained models indicated that such features did not contribute to the classification accuracy as much as kinematic or geometric features.

Jerkovic et al. (2019) experimented with in-air handwriting features and multiclass linear discriminant analysis (cLDA) to differentiate between HCs, patients with PD and patients with atypical parkinsonism. Altogether, 43 subjects from Serbia were enrolled in the study. The task was to write a sentence in various scenarios, such as with or without looking at the monitor of the laptop during writing. Various kinematic features related to the in-air and on-surface trajectories were extracted. The combination of the on-surface and in-air features led to ACC of 86%, whereas a model trained only with in-air features had a slightly lower ACC of ~79%. The results led to the conclusion that kinematic features based on both the in-air and on-surface trajectories are equally important in the quantitative analysis of the handwriting of PD patients with various types of motor impairments.

Impedovo (2019) investigated the use of new velocity-based signal processing techniques for the advance diagnosis of PD based on the discrete Fourier transform (DFT; for assessing rapidity and fluency), sigma–lognormal modeling (SLM; for quantifying the constant tremor pattern of PD utilizing cepstrum properties) and the Maxwell–Boltzmann distribution (MBD; for modeling handwriting velocity profiles). In his work, he utilized online handwriting records from the PaHaW database. The newly proposed features were extracted together with conventional features (baseline; Impedovo et al., 2018) for all tasks in the database. When classification was performed using all features and all tasks, the newly proposed features were selected among the 10 best-performing features (ACC of 94%, SVM classifier) and outperformed the baseline features (ACC of 88% SVM classifier). The author was able to increase the HC/PD classification accuracy to 98% when using only the most suitable tasks (the Archimedean spiral, “lll” and the word “lektorka”).

In 2020, a study published by Aouraghe et al. (2020) introduced new kinematic features utilizing the discrete time wavelet transform (DTWT), the fast Fourier transform (FFT) and a Butter/adaptive filter in the diagnosis of PD. Altogether, 80 native Arabic speakers were enrolled. All of them wrote a particular segment of text on several lines. Additionally, to better predict the continuous degradation of PD handwriting, the output of the text task was segmented line by line using unsupervised K-means clustering (observing the variation in the x and y trajectories). All of the extracted features (new and conventional) were calculated for the whole text and for each segmented line separately (at least 4 lines). The best performance on the entire task corresponded to ACC of 85.7% (KNN classifier). The first line showed a slightly lower ACC of 78.6% when a decision tree (DT) classifier was used. The last line proved to be the most effective and discriminative segment in the study when utilizing the DWT (ACC of 92.9%). Segmentation proved to be a valid method, as the results confirmed the hypothesis that PD handwriting degradation, deterioration, and fatigue increase over time.

While the previous approaches relied on carefully designed handcrafted features, Vásquez-Correa et al. (2019) proposed directly feeding the raw captured signals and their derivatives into a 1D CNN. These authors utilized a rather small CNN with two convolutional and pooling layers. This procedure allowed ACC of 67% to be achieved in the classification of PD patients and HC subjects. The authors performed several experiments using only onset or offset data, constituting the 200 ms after the transition from on-surface to in-air movement or the transition from in-air to on-surface movement. However, this approach did not seem to improve the prediction accuracy.

There are also some other studies that confirm feasibility of the digitized spiral drawing for PD detection (Kamble et al., 2021) and PD stage classification (Zham et al., 2017).

### 2.2. Offline Handwriting

In contrast to approaches based on online handwriting, in which multiple modalities are available, offline handwriting approaches must rely on visual data only. This significantly limits the information that is available for classification. Moetesum et al. (2019) utilized a pretrained AlexNet CNN to extract features from images capturing handwriting samples. To further enhance the extraction of features and boost the performance, the authors combined three different types of image preprocessing techniques. With this approach, they obtained ACC of 76% on a single task from the PaHaW dataset and ACC of 83% when merging all tasks used for prediction.

Recently, Gazda et al. (2021) proposed the idea of multiple-fine-tuned CNNs for the classification of PD handwriting. Similar to the work of Moetesum et al. (2019), this approach relies on a pretrained CNN. However, Gazda et al. utilized datasets of handwriting samples to bridge the gap between the semantically different ImageNet dataset, which was used for network pretraining, and parkinsonian handwriting datasets. This approach enabled more efficient transfer learning, leading to ACC of 92.7% on the spiral drawing task from the NewHandPD dataset and ACC of 85.8% on the spiral drawing task from the PaHaW dataset.

Similarly, six pretrained CNNs (AlexNet, GoogLeNet, VGG16, VGG19, ResNet50, and ResNet101) were evaluated in Kamran et al. (2021) in terms of their performance on four different handwriting datasets. The obtained results strongly depended on the dataset, with the most challenging dataset being PaHaW. In this case, the classification accuracy was only 62.5%, compared to accuracies of over 90% for the HandPD, NewHandPD (Pereira et al., 2016) and Parkinson's Drawing (Zham et al., 2017) datasets.

Finally, the authors of Diaz et al. (2019) were able to merge the online and offline handwriting approaches by incorporating dynamic information into static images. This approach seemed to improve classification in cases where the task can be performed continuously without lifting the pen. The highest ACC of 75% was achieved using VGG as the feature extractor and a linear SVM as the classifier for a single drawing task (spiral). Further improvements were obtained by building an ensemble classifier based on the results from different tasks, yielding ACC of 86%.

For a better illustration, a summary of the related works is provided in Table 1. The overview of the related works based on online handwriting is in the upper part, and studies based on offline handwriting are in the bottom part of the table.

**Table 1:** Overview of the related works.

**References**	**Participants**	**Task**	**Features**	**Analysis**	**Results**
**Online handwriting**					
Impedovo et al. (2018)	29 PD, 36 HC	PaHaW–all	Kinematic, enthropy	GNB	ACC = 72.0%
Mucha et al. (2018b)	33 PD, 36 HC	PaHaW–all	FD-based kinematic	XGBoost	ACC = 97.1%
					EER = 23.6% (PD dur)
					EER = 12.5% (UPDRS V)
Rios-Urrego et al. (2019)	39 PD, 70 HC	Archimedean spiral	Kinematic, geometric	KNN	ACC = 83.3% (spiral)
		Short sentence	Spectral, non-linear	SVM	ACC = 75.0% (sentence)
Jerkovic et al. (2019)	33 PD, 10 HC	Various sentences	Kinematic	cLDA	ACC = 86.0%
Impedovo (2019)	37 PD, 38 HC	PaHaW–all	DFT, SLM, MBD	SVM	ACC = 94.0%
Aouraghe et al. (2020)	40 PD, 40 HC	Segment of text	DTWT, FFT	KNN	ACC = 85.7% (full text)
			Butter/adaptive filter	decision tree	ACC = 78.6% (first line)
Vásquez-Correa et al. (2019)	44 PD, 40 HC	14 drawings/writings	Original signal	1D CNN	ACC = 67.0%
**Offline handwriting**					
Moetesum et al. (2019)	37 PD, 38 HC	PaHaW–all	AlexNet CNN	SVM	ACC = 83.0%
Gazda et al. (2021)	64 PD, 71 HC	Archimedean spiral	Pre-trained CNN and transfer		ACC = 92.7% (NewHandPD)
	2 dataset		learning (ImageNet → PD dataset)		ACC = 85.8% (PaHaW)
Kamran et al. (2021)	PaHaW	Several drawings			ACC = 62.5% (PaHaW)
	HandPD		AlexNet, GoogLeNet, VGG16		ACC = 91.4% (HandPD)
	NewHandPD		VGG16, ResNet50, ResNet101		ACC = 98.4% (NewHandPD)
	PD Drawings				ACC = 90.0% (PD Drawings)
Diaz et al. (2019)	37 PD, 38 HC	PaHaW–all	VGG	SVM	ACC = 86.0%

*PD, Parkinson's disease; HC, healthy control; PaHaW, Parkinson's disease handwriting database (Drotar et al., 2016); FD, fractional order derivative; ACC, accuracy; EER, estimation error rate; PD dur, PD duration; GNB, gaussian naïve bayes classifier; xGBoost, extreme gradient boosting tree; KNN, K-nearest neighbors; SVM, support vector machine; cLDA, multi-class linear discriminant analysis; CNN, convolution neural network; ResNet, residual neural network; VGG, very deep CNN; DFT, discrete fourier transformation; SLM, sigma-lognormal model; MBD, maxwell-boltzmann distribution; DTWT, discrete time wavelet transform; FFT, fast Fourier transform; UPDRS V, UPDRS, part V: Hoehn and Yahr scale (Fahn and Elton, 1987)*.

## 3. Materials and Methods

### 3.1. Dataset

**Figure 1:**
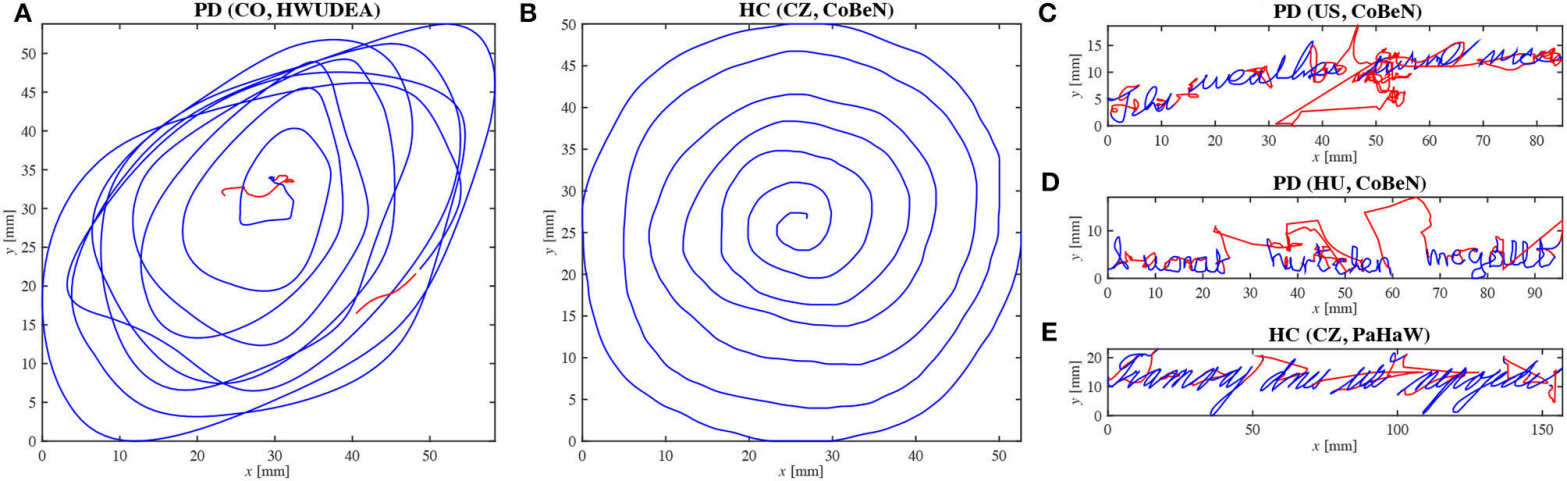
Selected samples from the multilingual dataset (blue line – on-surface movement; red line – in-air movement). **(A)** Spiral drawing (PD patient); **(B)** Spiral drawing (HC); **(C)** English sentence (PD patient) “The weather turned nice”; **(D)** Hungarian sentence (PD patient) “A vonat hirtelen megállt”; **(E)** Czech sentence (HC) “Tramvaj dnes už nepojede”.

In total, 143 patients with PD (71 female and 72 male; mean age 66.32±10.79 years) and 151 HCs (86 female and 65 male; mean age 64.79±9.90 years) were enrolled in several geographical locations: the Czech Republic (CZ), Hungary (HU), the United States of America (US), and Colombia (CO). A corresponding multilingual dataset was created by fusing the following databases: PaHaW (Drotar et al., 2016), CoBeN (acquired under the Marie Skłodowska-Curie grant agreement no. 734718), and HWUDEA (Castrillon et al., 2019; Rios-Urrego et al., 2019). In the case of the PaHaW database, the participants performed 9 tasks (e.g., Archimedean spiral, letters, syllables, words, sentence) on A4 paper that was laid down and fixed to a digitizing tablet (Wacom Intuos 4M, with a sampling frequency of *f*_s_ = 133Hz). A special Wacom inking pen was used to provide immediate visual feedback, i.e., simulating classical pen-and-paper writing/drawing. The participants enrolled for the acquisition of CoBeN underwent a protocol consisting of 8 tasks (e.g., connecting dots, overlapping pentagons, Archimedean spiral, sentences) using a similar paper–tablet setup; however, in this case, the data were recorded by a Wacom Intuos Pro L (*f*_s_ = 133Hz). Finally, the HWUDEA database was acquired by employing a Wacom Cintiq 13HD Touch display tablet (*f*_s_ = 180Hz). In total, 17 tasks were recorded for each participant (e.g., spring, alphabet, sentence, Archimedean spiral, house drawing). Although the databases were collected following different protocols, all of them share two tasks: the Archimedean spiral drawing task and a sentence writing task. Selected samples can be seen in Figure 1.

Demographic data with respect to each of the two tasks shared among all databases are reported in Table 2. Unfortunately, the databases are not annotated with the same clinical information (e.g., the CoBeN–HU dataset contains only information about sex and age); nevertheless, to provide at least limited insight into the characteristics of the PD patients, we summarize the available metadata in Table 3. None of the participants had a history or the presence of any psychiatric symptoms, cognitive impairment, or any disease affecting the central nervous system (other than PD in the PD cohort). All PD patients were diagnosed based on the diagnostic criteria for PD (Postuma et al., 2016). They were well-compensated on their stable dopaminergic medication and without major motor fluctuations or dyskinesias [they were examined while on their regular dopaminergic medication (ON state) ~1 h after the L-dopa dose]. All subjects signed informed consent forms. The study was approved by the relevant local ethics committees.

**Table 2:** Demographic characteristics.

**Dataset**	**Language**	**PD (*N*; female)**	**PD (*N*; male)**	**PD (age)**	**HC (*N*; female)**	**HC (*N*; male)**	**HC (age)**
**Archimedean spiral**							
PaHaW	CZ	18	15	69.21 ± 11.10	17	19	62.50 ± 11.70
CoBeN	CZ	6	13	66.48 ± 7.77	30	10	67.04 ± 6.07
CoBeN	US	3	6	68.56 ± 4.07	9	3	72.50 ± 8.37
CoBeN	HU	2	7	66.00 ± 9.96	7	5	64.92 ± 5.30
HWUDEA	CO	41	28	64.42 ± 11.85	22	27	62.69 ± 11.34
**Sentence**							
PaHaW	CZ	19	18	69.32 ± 10.97	18	20	62.42 ± 11.39
CoBeN	CZ	6	13	66.48 ± 7.77	30	9	67.21 ± 6.05
CoBeN	US	3	6	68.56 ± 4.07	9	3	72.50 ± 8.37
CoBeN	HU	2	6	65.88 ± 10.64	7	5	64.92 ± 5.30
HWUDEA	CO	13	4	63.88 ± 7.61	5	5	70.20 ± 10.67

**Table 3:** Clinical characteristics of the PD patients.

**Dataset**	**Language**	**Duration of PD [years]**	**LED [mg/day]**	**UPDRS III**	**UPDRS V**
PaHaW	CZ	8.38 ± 4.80	1,432.19 ± 704.78	–	2.27 ± 0.85
CoBeN	CZ	4.00 ± 4.15	568.33 ± 508.03	7.00 ± 1.41	–
CoBeN	US	–	333.12 ± 240.40	–	–
CoBeN	HU	–	–	–	–
HWUDEA	CO	10.56 ± 11.16	–	36.78 ± 19.63	2.38 ± 0.61

*LED, L-dopa equivalent daily dose (Lee et al., 2010); UPDRS III, Unified Parkinson's Disease Rating Scale, part III: motor examination (Fahn and Elton, 1987); UPDRS V, UPDRS, part V: Hoehn and Yahr scale (Fahn and Elton, 1987)*.

### 3.2. Scenarios

We define three main scenarios to analyze the effect of linguality on the classification of PDYS:

1. Single language—In this scenario, we consider datasets for every language separately. As such, there are four different models: HU, US, CO, and CZ (the Czech dataset is created by merging the PaHaW and CoBeN datasets). In this scenario, each classification model is trained and tested on a dataset consisting of data samples that all come from the same language. This scenario is considered to correspond to internal model validation because the linguality of the datasets is not considered; rather, the robustness of the features is evaluated at the per-dataset level.
2. Leave one language out—In this scenario, the influence of different languages on the classification performance is evaluated by training each model on three out of four datasets and testing it on the remaining dataset. With this approach, we aim to investigate the effect of transferring knowledge between datasets coming from different language sources. We refer to this scenario as the leave-one-language-out scenario. This scenario is considered to correspond to external model validation because the multilinguality of the data is taken into account, i.e., the validation samples come from a different geographical location, as recommended in the TRIPOD guidelines (Collins and Moons, 2019).
3. All languages combined—In the last scenario, we combine all datasets of different languages into one complete dataset to evaluate the performance of the features on the mixed dataset.

### 3.3. Feature Extraction

Although the individual databases were acquired using different devices, all of them recorded the following information (time series): the x and y positions (*x*[*n*] and *y*[*n*]), the timestamp (*t*[*n*]), a binary variable (*b*[*n*]) taking values of 0 for in-air movement (i.e., movement of the pen tip up to 1.5 cm above the tablet's surface) and 1 for on-surface movement (i.e., movement of the pen tip on the paper), the pressure exerted on the tablet's surface during writing (*p*[*n*]), the pen tilt (*a*[*n*]), and the pen azimuth (*az*[*n*]). First, we preprocessed the recordings for unit unification (e.g., we expressed the x and y positions in millimeters, time in seconds, etc.) and resampling [we resampled all signals to *f*_s_ = 133Hz employing a finite impulse response (FIR) antialiasing low-pass filter]. Subsequently, we parameterized the signals employing the previously mentioned baseline and CNN-based features.

#### 3.3.1. Baseline Features

To establish a good baseline for the evaluation of the CNN-based features, we consulted several recent articles and reviews (Impedovo and Pirlo, 2018; De Stefano et al., 2019; Vessio, 2019) and extracted the handcrafted features that are most commonly used for the quantitative assessment of PD dysgraphia. These features can be divided into six groups:

1. Temporal—duration of writing (DUR), ratio of the on-surface/in-air durations (DURR), duration of strokes (SDUR), and ratio of the on-surface/in-air stroke durations (SDURR)
2. Spatial—width (WIDTH), height (HEIGHT), and length (LEN) of the whole product as well as those of its individual strokes, i.e., stroke width (SWIDTH), height (SHEIGHT), and length (SLEN)
3. Kinematic—velocity (VEL), angular velocity (AVEL), and acceleration (ACC)
4. Dynamic—pressure (PRESS), tilt (TILT), and azimuth (AZIM)
5. Spiral-specific (San Luciano et al., 2016; Cascarano et al., 2019)—first-order smoothness of spiral (1stSm), second-order smoothness of spiral (2ndSm), spiral tightness (TGHTNS), first-order zero-crossing rate of spiral (1stZC), second-order zero-crossing rate of spiral (2ndZC), degree of spiral drawing severity (DoS), mean drawing speed of spiral (MDS), variability of spiral width (SWVI), and spiral precision index (SPI)
6. Other—number of interruptions or pen elevations (NINT), relative number of interruptions (RNINT), number of on-surface interstroke intersections (NIEI), relative number of on-surface interstroke intersections (RNIEI), number of on-surface intrastroke intersections (NIAI), relative number of on-surface intrastroke intersections (RNIAI), total number of on-surface intrastroke intersections (TNIAI), relative total number of on-surface intrastroke intersections (RTNIAI), relative number of changes in velocity profile (RNCV), relative number of changes in pressure profile (RNCP), relative number of changes in tilt profile (RNCT), and relative number of changes in azimuth profile (RNCA)

The spatial, temporal, and kinematic features were extracted from both the on-surface and in-air movements. In addition, the kinematic features were also analyzed for the horizontal and vertical projections of the movements. Features that are represented by time series were transformed into scalar values using the median, interquartile range (iqr), nonparametric coefficient of variation (ncv; defined as iqr/median), and slope by applying the Theil–Sen estimator (slope). In the case of the kinematic time series, we also calculated the 95th percentile (95p).

For each feature, we use the following notation: *INF: DIR-FN (HL)*, where *INF* denotes the processed information (ON for on-surface, AIR for in-air, PRESS for pressure, TILT for tilt, and AZIM for azimuth), *DIR* denotes the direction (H for horizontal and V for vertical), *FN* is the feature name, and *HL* is the statistic used for the transformation.

#### 3.3.2. CNN-Based Features

Over the past decade, CNNs have demonstrated outstanding capabilities on various tasks, such as image recognition, medical image analysis, and handwriting recognition. Multiple state-of-the-art models exist, with a typical structure consisting of an input layer, a mix of convolutional and pooling layers, and one output layer. Deeper networks often produce better results than shallower ones; on the other hand, they have multiple times more parameters and require more data for training, especially when compared to traditional machine learning models. To overcome this problem, transfer learning techniques have been proposed.

The idea behind transfer learning is to take advantage of the features of a CNN trained on one task and use them for another task. Given a source domain *D*_*s*_, a corresponding task *T*_*s*_, a target domain *D*_*t*_, and the corresponding task *T*_*t*_, where *D*_*s*_≠*D*_*t*_ and *T*_*s*_≠*T*_*t*_, the goal of transfer learning is to reduce the error of the target predictive function *f*_*t*_(.) in *D*_*t*_. For transfer learning, two main paradigms exist. The first is called fine tuning, in which a neural network or at least part of the neural network is retrained, thus changing the weights of the layers. In the second approach, a CNN is used to extract features. In the feature extraction model, the weights trained on the source task are frozen, and the corresponding representations are applied in the target task.

In case of CNN-based features we render images from data captured by the digitizing tablet. Specifically, we use only the x and y positions (*x*[*n*] and *y*[*n*]). To extract CNN-based features, we employed the state-of-the-art CNN known as VGG16 (Simonyan and Zisserman, 2014), pretrained on the ImageNet dataset (Russakovsky et al., 2015). The VGG16 is well-known architecture that is still being frequently used thanks to its relative simplicity. The input images were resized to 224 ×224 by nearest-neighbor interpolation. We extracted features from the last convolutional layer in the VGG16 network. The extracted features capture abstract representations of the processed input image. Features were classified by CNN head consisting of fully connected layer and output layer.

### 3.4. Machine Learning

For the handcrafted features, we built binary classification models using an ensemble extreme gradient boosting algorithm known as XGBoost (Chen and Guestrin, 2016). The reason behind using such an advanced nonlinear classifier is to search for complex nonlinear patterns in a feature set composed of rather simple feature representations. To build models with the optimal hyperparameters, we applied a randomized search strategy to optimize the following set of hyperparameters: the learning rate [0.001, 0.01, 0.1, 0.2, 0.3], γ [0, 0.05, 0.10, 0.15, 0.20, 0.25, 0.5], the maximum tree depth [6, 8, 10, 12, 15], the fraction of observations to be randomly sampled for each tree (subsample ratio) [0.5, 0.6, 0.7, 0.8, 0.9, 1.0], the subsample ratio for the columns at each level [0.4, 0.5, 0.6, 0.7, 0.8, 0.9, 1.0], the subsample ratio for the columns when constructing each tree [0.4, 0.5, 0.6, 0.7, 0.8, 0.9, 1.0], the minimum sum of the weights of all observations required in a child node [0.5, 1.0, 3.0, 5.0, 7.0, 10.0], and the balance between positive and negative weights [1, 2, 3, 4].

In contrast, the binary classification models for the CNN-based features were built using L2-regularized logistic regression (LR), also known as ridge regression. The reason behind using this much simpler linear classifier is the assumption that the underlying nonlinear representations are already captured by the CNN-extracted features. In addition, features extracted from convolutional layers tend to have very high dimensionality, and thus, using a simpler classifier minimizes the chance of overfitting and maximizes the computational efficiency. To find the optimal parameters of the LR classifier, we searched through the various settings for the regularization parameter C given by the following set: [0.001, 0.01, 0.1, 1, 10, 100, 1000].

The randomized search was conducted 500 times. In both cases, the objective of the hyperparameter search was to optimize the balanced accuracy score (BACC; described in more detail along with other evaluation scores below) *via* stratified five-fold cross-validation with five repetitions (the five-fold cross-validation scheme was chosen as a reasonable compromise between the numbers of samples in the training and validation folds, i.e., to provide the classifier with sufficient training samples while also testing its performance on a representative subset of the overall sample size).

Finally, the trained classification models were evaluated on a per-scenario basis: (a) single language—in this scenario, we conducted stratified five-fold cross-validation with five repetitions; (b) leave one language out—in this scenario, we tested the performance of each trained classifier on the remaining dataset that was not present in the training data; and (c) all languages combined—in this scenario, we again employed stratified five-fold cross-validation with five repetitions. Only one sample of Archimedean spiral or sentence was available from each subject. Therefore, all decisions are based on a per subject basis. The classification test performance was established using the following well-known and widely used classification metrics: BACC, sensitivity (SEN), specificity (SPE), and F1 score.

## 4. Results

### 4.1. Single-Language Scenario

The classification performance of the models trained in this scenario is summarized in Table 4. First, we trained and tested the classification models using the spiral drawing task. The highest BACC values of 82% (handcrafted features) and 77% (CNN-based features) were achieved for the US dataset. These accuracies are notably higher than those achieved for the other datasets, which indicates that the US samples most likely carry certain recognizable patterns of PD related to the graphomotor difficulties manifested during spiral drawing. With respect to the comparison between the handcrafted and CNN-based features, the results show similar trends, with both types of features yielding the highest accuracy on the US dataset and quite similar results on the other datasets. More specifically, the CNN-based features outperformed the handcrafted features on the CZ dataset (BACCs of 64 vs. 59%) as well as on the CO dataset (BACCs of 61 vs. 59%) but yielded less accurate predictions on the US dataset. This shows that CNNs, even when provided with visual information only, can be competitive with handcrafted features on the spiral drawing task. However, there is one exception. From the performance of the CNN-based features on the HU dataset, it is evident that this model failed to provide reasonable predictions (BACC of 48% with the CNN-extracted features as opposed to BACC of 64% with the handcrafted features).

**Table 4:** Classification performance in the single-language scenario.

**Language**	**Features**	**BACC**	**F1**	**SEN**	**SPE**
**Spiral drawing**					
CZ	Handcrafted	0.59 ± 0.08	0.590.07	0.82 ± 0.12	0.36 ± 0.14
	CNN	0.64 ± 0.03	0.65 ± 0.05	0.65 ± 0.09	0.65 ± 0.06
CO	Handcrafted	0.59 ± 0.12	0.72 ± 0.07	0.81 ± 0.09	0.37 ± 0.23
	CNN	0.61 ± 0.02	0.62 ± 0.02	0.62 ± 0.03	0.62 ± 0.02
HU	Handcrafted	0.64 ± 0.17	0.61 ± 0.20	0.72 ± 0.29	0.57 ± 0.34
	CNN	0.48 ± 0.03	0.52 ± 0.13	0.52 ± 0.16	0.52 ± 0.12
US	Handcrafted	0.82 ± 0.18	0.77 ± 0.28	0.84 ± 0.31	0.81 ± 0.23
	CNN	0.77 ± 0.02	0.77 ± 0.07	0.77 ± 0.11	0.77 ± 0.08
**Sentence writing**					
CZ	Handcrafted	0.66 ± 0.08	0.62 ± 0.08	0.64 ± 0.10	0.69 ± 0.12
	CNN	0.65 ± 0.04	0.66 ± 0.04	0.66 ± 0.04	0.66 ± 0.05
CO	Handcrafted	0.56 ± 0.18	0.72 ± 0.19	0.83 ± 0.22	0.28 ± 0.29
	CNN	0.50 ± 0.08	0.54 ± 0.07	0.54 ± 0.08	0.54 ± 0.09
HU	Handcrafted	0.75 ± 0.18	0.65 ± 0.30	0.82 ± 0.34	0.59 ± 0.34
	CNN	0.50 ± 0.06	0.48 ± 0.08	0.48 ± 0.10	0.48 ± 0.08
US	Handcrafted	0.65 ± 0.20	0.54 ± 0.28	0.58 ± 0.34	0.73 ± 0.32
	CNN	0.70 ± 0.04	0.70 ± 0.06	0.70 ± 0.08	0.70 ± 0.05

*BACC, balanced accuracy; F1, F1 score; SEN, sensitivity; SPE, specificity*.

**Figure 2:**
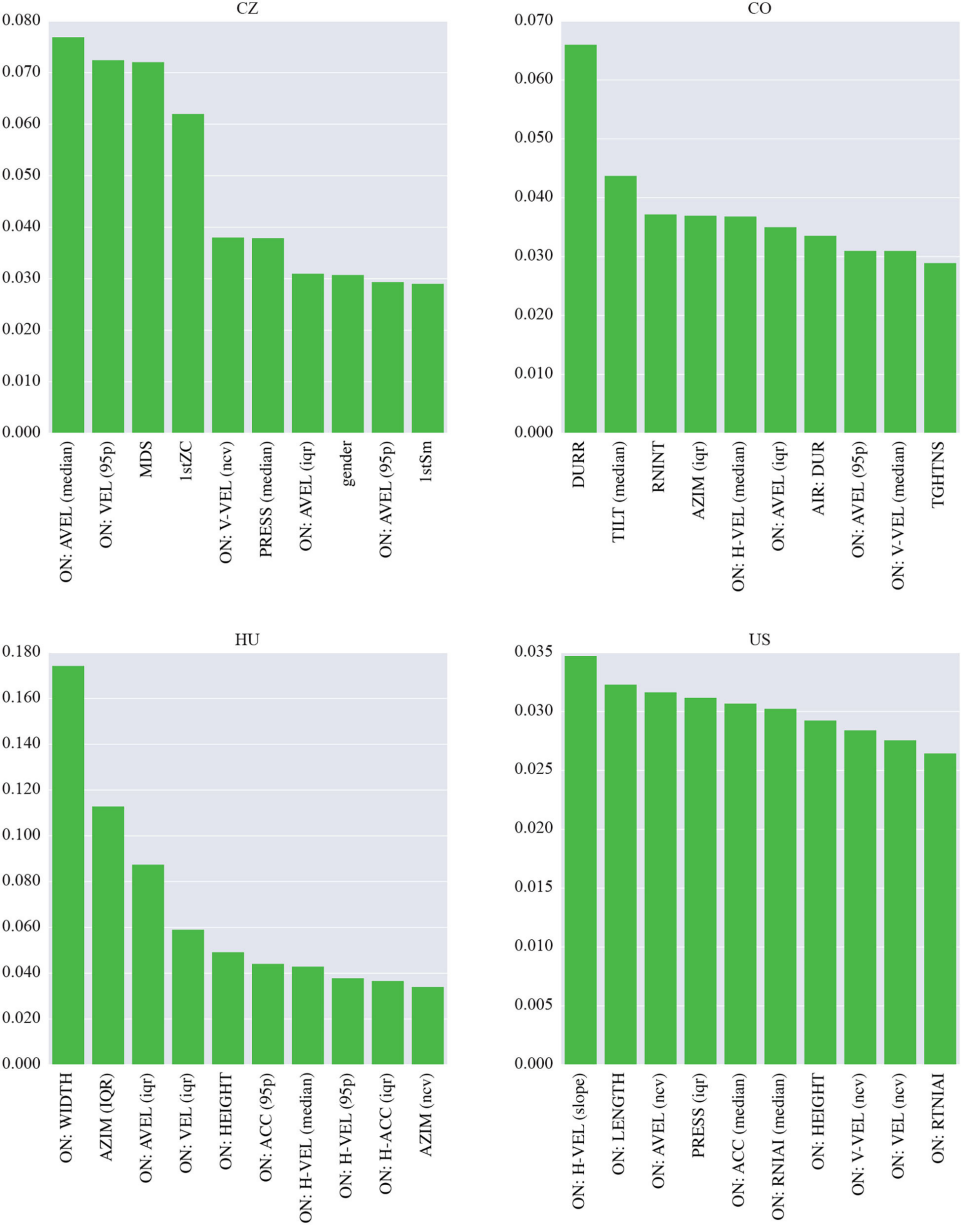
Importance of the features used in the models in the single-language scenario (spiral drawing task).

To interpret the machine learning models, we investigated the top ten most important features (see Figure 2). In the CZ dataset, most of these features are derived from the on-surface angular velocity. Other kinematic features are based on the on-surface velocity and the mean drawing speed of the spiral. Finally, the zero-crossing rate of the spiral, the pressure and the spiral smoothness all show some importance. The most important feature in the CO dataset is the ratio between the on-surface and in-air durations. It is followed by the relative number of interruptions and by the tilt-based and azimuth-based parameters. The important feature set also contains the in-air duration and spiral tightness. The rest of the features are based on the angular velocity and horizontal/vertical velocity. The most important set of features for the HU model contains two spatial parameters, width and height. The variation in azimuth plays an important role as well. Finally, the majority of the important features are kinematic (angular velocity, velocity, and acceleration). These features are also important in the US database. In addition, some spatial parameters (length and height), the pressure and the intraspiral intersections are identified as important.

Second, we evaluated the models for the sentence writing task in the same scenario. There are a few interesting points to note. First, prediction fails on the CO dataset for both types of features (BACC of 56% with the handcrafted features and BACC of 50% with the CNN-based features). The reason is most likely the small sample size; in the CO data, there are only 27 sentences, compared to the 118 spirals used in the previous experiment. Next, the model utilizing the handcrafted features clearly outperformed the model based on the CNN features on the HU dataset (BACC of 75% with the handcrafted features and BACC of 50% with the CNN-based features) and yielded slightly more accurate predictions on the CZ dataset (BACC of 66% vs. BACC of 65%). This is to be expected since for CNN-based features, a larger sample size is probably needed to learn the underlying patterns from a given sentence; compared with spiral drawing, sentence writing is much less restricted in terms of what the final handwritten product should look like. Finally, even though the US dataset contains spirals and sentences from the same patient group, the classification accuracy is significantly lower for the sentence writing task than for the spiral drawing task. Quite surprisingly, the CNN-extracted features outperformed the handcrafted features for the US cohort (BACCs of 70 vs. 65%).

**Figure 3:**
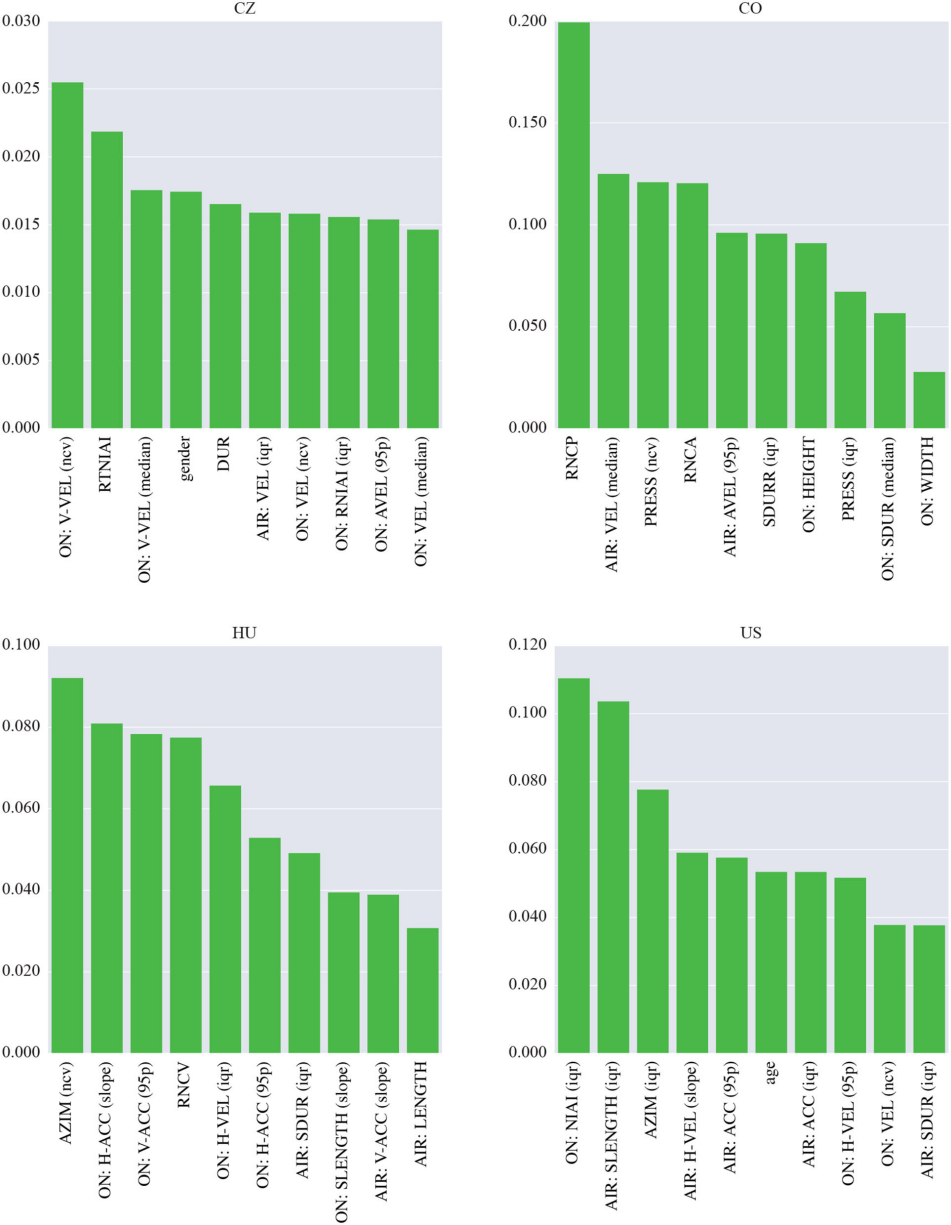
Importance of the features used in the models in the single-language scenario (sentence writing task).

Regarding the interpretation of the models shown in Figure 3, the most important features in the CZ dataset are based on the on-surface velocity, more specifically on its median and variation. In addition, the two highest-ranked velocity-based parameters are derived from the vertical projection. The most important feature set also contains the duration and number of intrastroke intersections. The most important feature in the CO dataset is the relative number of changes in the pressure profile, and two other pressure-based parameters (range and variation) were also selected. The last dynamic parameter is the number of changes in the azimuth profile. Regarding kinematic features, the set contains the in-air velocity and angular velocity. The stroke duration and spatial features such as width and height also play important roles. In the HU dataset, the most important feature is the variation in azimuth. Other significant features include the on-surface and in-air acceleration, and the on-surface horizontal velocity and the relative number of changes in the velocity profile are also important. Temporal features are represented by the in-air stroke duration. Finally, two important spatial parameters are identified: the on-surface stroke length and the overall length of the in-air movement. The three most important features in the US dataset are the number of on-surface intrastroke intersections, the in-air stroke length and the range of the azimuth. These are followed by mainly kinematic parameters, i.e., the in-air horizontal velocity, in-air acceleration, and on-surface velocity (including its horizontal projection). In terms of temporal features, the set also contains the in-air stroke duration.

**Figure 4:**
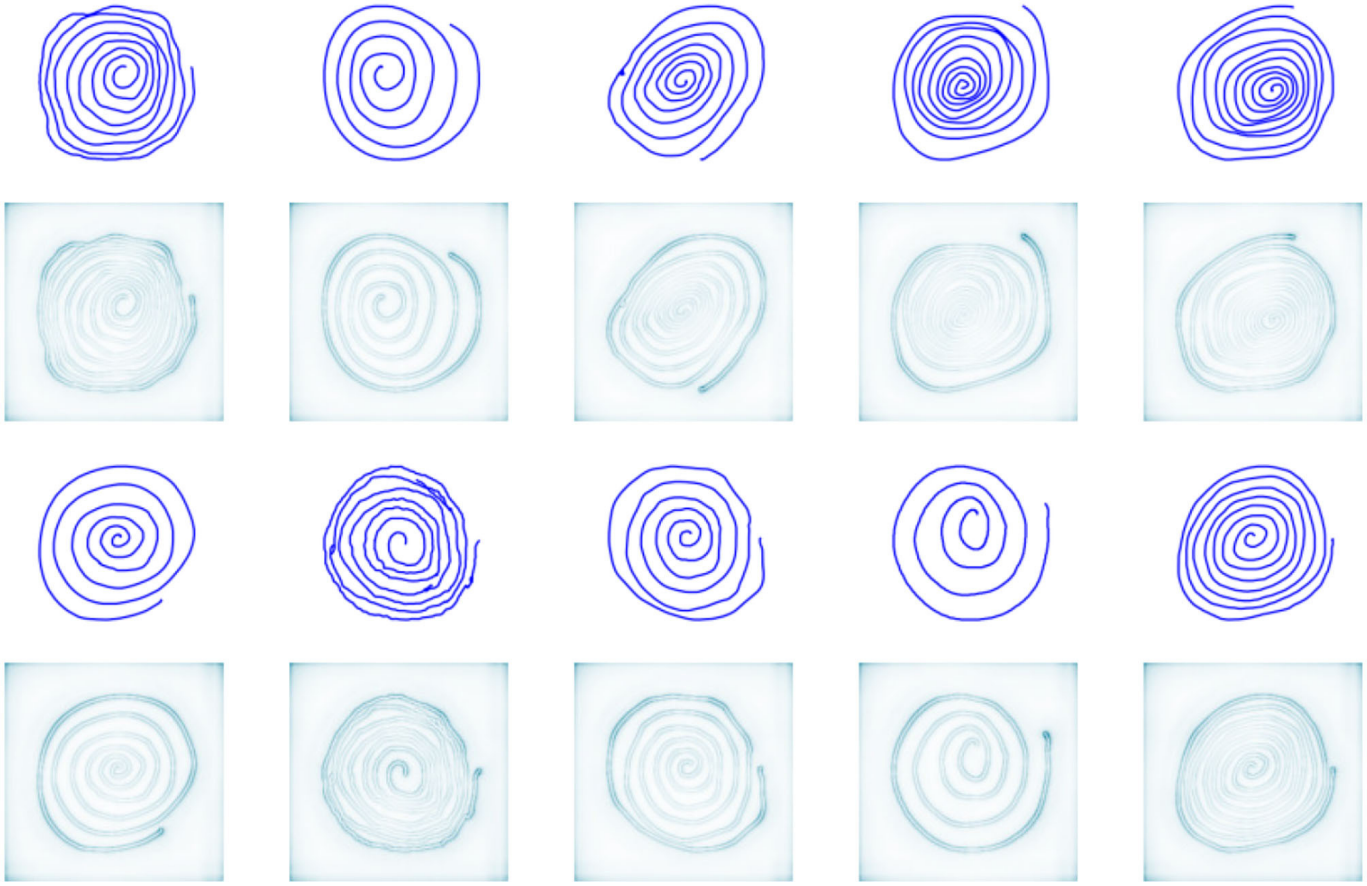
Relevance maps for ten Archimedean spirals (two random samples from each dataset are depicted).

**Figure 5:**
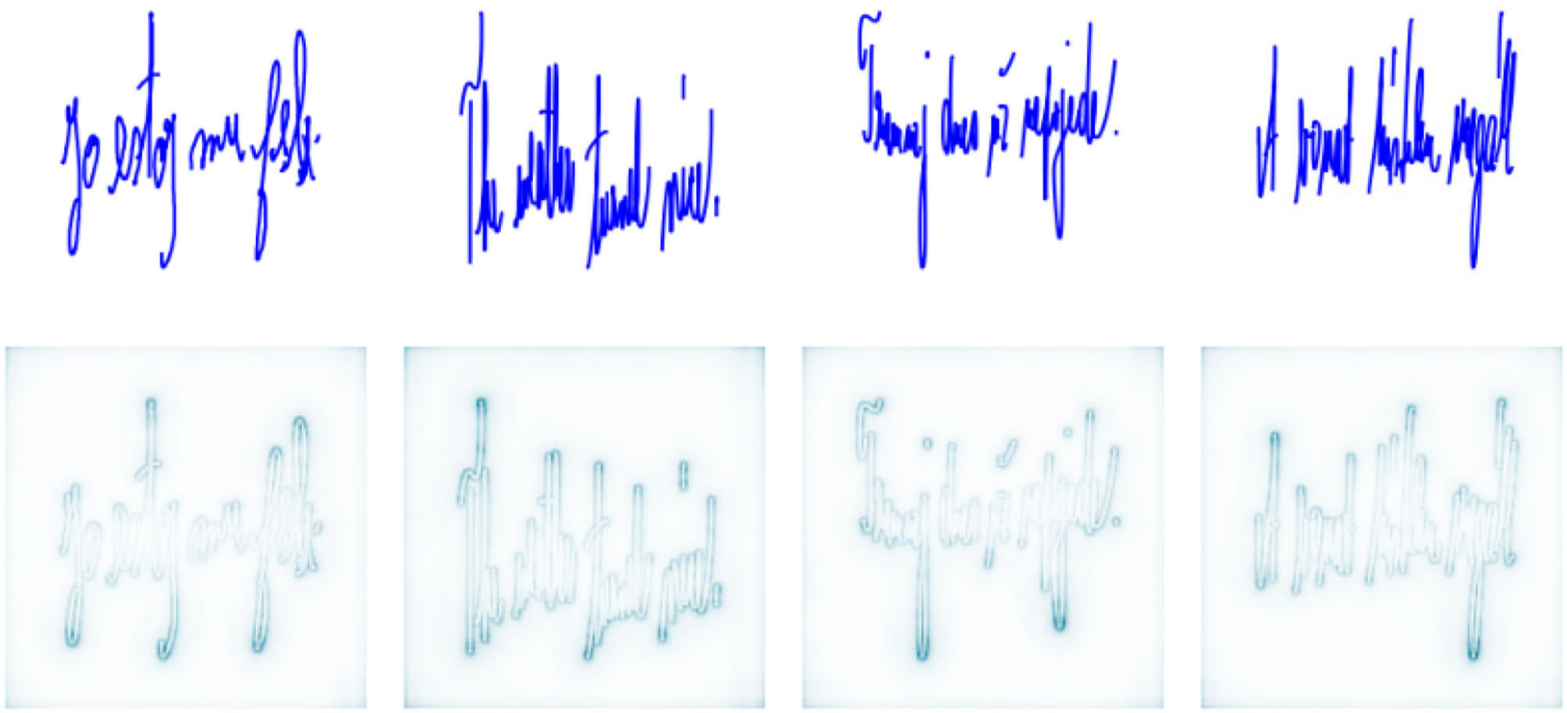
Relevance maps for four sentences (one random sample is depicted for each language).

The interpretation of CNN decisions is not straightforward since CNN models work in a black-box manner. We employ deep Taylor decomposition (Montavon et al., 2017) to gain a better understanding of the decisions made. Deep Taylor decomposition generates relevance maps illustrating the importance of single pixels in images. Figures 4, 5 show the relevance maps for ten spirals and four sentence writing samples, illustrating the pixels that were considered the most relevant for CNN-based feature extraction. Note that all figures that were used as CNN input were rendered at a resolution of 244 ×244 pixels. This resolution is optimal for the pretrained VGG network, but it created some deformation of the handwriting in the sentence writing task. This might have produced suboptimal results; however, using different resolutions would have required training the whole network from scratch, which would have been incompatible with the intention of this study.

### 4.2. Leave-One-Language-Out Scenario

The classification performance of the models trained in this scenario is summarized in Table 5. Naturally, the native language of a participant exerts no influence on the spiral drawing task; however, we can still investigate how the models performed on external validation datasets. When the CZ dataset was used as the test set, BACC degraded from 59 to 54% and from 64 to 45% for the handcrafted and CNN-based features, respectively. In contrast, in the case of the CO test set, BACC with the handcrafted features decreased from 59 to 50%, while the performance of the CNN-based features slightly improved, specifically from 61 to 63%. In the case of the HU test set, BACC with the handcrafted features similarly degraded from 64 to 56%, but interestingly, when the CNN-extracted features were used, the classification performance improved from 48 to 71%, even higher than in the internal model validation in the previous experiment. This can be explained by the fact that the HU dataset is quite small, so the model was not able to learn well from data coming from the HU dataset only. Finally, the prediction performance on the US test set, which yielded optimistic results in the single-language scenario, decreased dramatically. For the handcrafted features, BACC decreased from 82 to 65%, and for the CNN-based features, the model completely failed to generalize, as BACC decreased from 77 to only 38%. This shows that the pattern responsible for the high classification accuracy in the internal model validation is most likely not present (or is less prominent) in the other datasets.

**Table 5:** Classification performance in the leave-one-language-out scenario.

**TRAIN**	**TEST**	**Features**	**BACC**	**F1**	**SEN**	**SPE**
**Spiral drawing**						
CO+HU+US	CZ	Handcrafted	0.54	0.51	0.62	0.46
		CNN	0.45	0.41	0.48	0.42
CZ+HU+US	CO	Handcrafted	0.50	0.74	1.00	0.00
		CNN	0.63	0.62	0.54	0.71
CZ+CO+US	HU	Handcrafted	0.56	0.47	0.44	0.67
		CNN	0.71	0.67	0.67	0.75
CZ+CO+HU	US	Handcrafted	0.65	0.67	0.88	0.41
		CNN	0.38	0.32	0.33	0.42
**Sentence writing**						
CO+HU+US	CZ	Handcrafted	0.63	0.68	0.78	0.48
		CNN	0.54	0.58	0.80	0.29
CZ+HU+US	CO	Handcrafted	0.59	0.30	0.18	1.00
		CNN	0.51	0.72	0.82	0.20
CZ+CO+US	HU	Handcrafted	0.67	0.64	0.59	0.75
		CNN	0.60	0.46	0.38	0.83
CZ+CO+HU	US	Handcrafted	0.71	0.67	0.59	0.83
		CNN	0.63	0.46	0.33	0.92

*TRAIN, training dataset; TEST, test dataset; BACC, balanced accuracy; F1, F1 score; SEN, sensitivity; SPE, specificity*.

Regarding the sentence writing task, the language does exert an influence, and it is therefore important to look at the differences in the classification performance achieved in the internal and external validations. When the CZ dataset was used as the test set, BACC decreased from 66 to 63% and from 65 to 54% for the handcrafted and CNN-based features, respectively. In the case of the CO test set, BACC decreased from 56 to 50% for the handcrafted features and from 59 to 51% for the CNN-extracted features. With respect to the HU test set, BACC degraded from 75 to 67% for the handcrafted features but improved from 50 to 60% for the CNN-based features. This is consistent with the results of the spiral drawing task, for which the classifier based on the CNN-extracted features needed more data for training. In the case of the US test set, BACC improved for the handcrafted features, from 65 to 70%, but decreased for the CNN-extracted features, from 71 to 63%.

Interestingly, the classifiers utilizing the CNN-based features extracted from the spiral drawing task either outperformed those trained on the handcrafted features or failed to generalize, whereas the classifiers based on the handcrafted features extracted from the sentence writing task yielded higher classification accuracy in all four experiments (with different combinations of training and test datasets). This was to be expected since in the latter case, the models were trained on sentences with orthography different from that in the test set. These findings confirm the hypothesis that the handcrafted features designed by domain experts are more robust than automatically extracted CNN-based features in cases in which different visual patterns are to be evaluated.

### 4.3. Scenario With All Languages Combined

In the last scenario, we combined the samples from all languages together to create a single heterogeneous dataset. The classification performance of the models trained in this scenario is summarized in Table 6. In the case of the spiral drawing task, the handcrafted features and CNN-based features show very similar performance, achieving 60% accuracy. The hypothesis that CNN-based features are more sensitive to the visual orthography of the sentence writing task is also confirmed by this last scenario, as the classifier based on handcrafted features outperformed the one trained on CNN-extracted features, achieving almost 70% accuracy (although in this case, the difference was much less prominent).

**Table 6:** Classification performance in the scenario with all languages combined.

**Task**	**Features**	**BACC**	**F1**	**SEN**	**SPE**
Spiral	Handcrafted	0.60 ± 0.06	0.63 ± 0.06	0.73 ± 0.10	0.48 ± 0.07
	CNN	0.60 ± 0.01	0.61 ± 0.02	0.61 ± 0.04	0.61 ± 0.04
Sentence	Handcrafted	0.69 ± 0.05	0.65 ± 0.07	0.61 ± 0.09	0.78 ± 0.07
	CNN	0.66 ± 0.01	0.67 ± 0.01	0.67 ± 0.03	0.67 ± 0.03

*BACC, balanced accuracy; F1, F1 score; SEN, sensitivity; SPE, specificity*.

## 5. Discussion

We compared the results of two different approaches to feature extraction: handcrafted features and features extracted by a CNN. In the case of the handcrafted features, we utilized a set of baseline features that are frequently used for handwriting analysis. We focused mainly on temporal, spatial, kinematic, and dynamic features, and we did not employ any advanced nonconventional features. Similarly, in the case of the CNN-extracted features, we used a pretrained VGG network to extract the features, although propositions have already emerged for improving the methodologies applied to diagnose PD from offline handwriting (Moetesum et al., 2019; Gazda et al., 2021). The motivations for this are two-fold. First, our aim was to establish baseline results that can be used as a reference in the future. Second, by using these baseline approaches, we could provide a fair comparison between the classification performance of handcrafted features and CNN-extracted features.

Regarding clinical interpretability, the models based on the Archimedean spiral drawing task mainly utilized kinematic features. This finding is reasonable because the cardinal symptoms of PD, such as rigidity, akinesia, and bradykinesia, have a significant impact on fine motor skills, including handwriting/drawing (Letanneux et al., 2014). Generally, PDYS is associated with reduced velocity (Ponsen et al., 2008; Rosenblum et al., 2013; Impedovo and Pirlo, 2018; De Stefano et al., 2019), which could occur more frequently than the most pronounced symptom, micrographia (Letanneux et al., 2014). Since the Archimedean spiral drawing task is a task in which subjects perform coordinated rotation, among the kinematic parameters, the angular velocity seems to play the most important role in the differentiation of PD/HC subjects.

Interestingly, features specifically designed for the assessment of Archimedean spiral drawing in PD patients (San Luciano et al., 2016; Cascarano et al., 2019; such as the smoothness of the spiral, the spiral tightness, the variability of the spiral width, and the spiral precision index) were not as important as we initially assumed. Similar to the dynamic features (e.g., pressure, tilt, azimuth), spatial features (width, height, length), and temporal features (duration), they were important only in some specific datasets.

Concerning the clinical interpretability of the models based on the sentence writing task, except for the CO database, all models were again based mainly on kinematic features, mostly extracted from the on-surface movement. In terms of projection, kinematic deficits were observed in both the horizontal and vertical movements. Nevertheless, in the largest database (CZ), deficits mainly dominated in the vertical projection. Kushki et al. (2011) reported that the finger system (which is mainly involved in vertical movement) is more affected by muscular fatigue than the wrist system (which controls horizontal movement). From an anatomical point of view, vertical movement requires coordinated movement and finer flexions/extensions of more joints (interphalangeal and metacarpophalangeal), i.e., it is more complex than ulnar abductions of the wrist (Van Galen, 1991; Dounskaia et al., 2000), and we assume this to be the reason why kinematic deficits were more strongly observed in this direction. This finding could also be somehow linked with progressive/consistent vertical micrographia, i.e., progressive/consistent reduction in letter amplitude (Thomas et al., 2017). However, this hypothesis requires further research because some studies suggest that the horizontal version of micrographia is even more common than the vertical version (Thomas et al., 2017).

Interestingly, except for the CZ database, the azimuth also played a significant role, more specifically its variation and range. We have identified one publication in which the authors advantageously utilized azimuth-based features in the semisupervised modeling of PDYS (Ammour et al., 2020). We assume that tremor could lead to improper coordination of the upper extremities, which could manifest as unstable azimuth features during the process of handwriting.

Temporal features (the duration of the whole process or of individual strokes) additionally played an important role in all models. In some studies, duration has not been found to be useful for discriminating between PD patients and HCs because although patients with PD write slowly, they also write smaller letters and thus ultimately spend the same time on, e.g., copying a sentence (Letanneux et al., 2014; Vessio, 2019). Nevertheless, in our case, with a few exceptions, spatial parameters were not found to be important in PDYS modeling.

Although it has been reported that PD patients generally apply less pressure (Rosenblum et al., 2013), we observed an important role of pressure-based features only in the CO model. Since only the CO database was recorded using the Wacom Cintiq tablet, the question arises of whether the corresponding discriminative power is associated solely with the disease or whether it is somehow enhanced by writing on a display.

In contrast to conventional shallow machine learning models, deep CNN models are quite challenging to interpret because of the dimensionality and complexity involved. However, as mentioned in the previous sections, we employed deep Taylor decomposition (Montavon et al., 2017) to create relevance maps illustrating the pixels that were considered most relevant for CNN-based feature extraction.

Regarding the spiral drawing task, as seen from Figure 4, the pixels that were assigned the highest weight for decisions lay along the outline of the drawn image. This indicates that the outer curve may convey information that can be explored to differentiate PD patients and HCs. We can hypothesize that this location in the spiral is strongly related to the shape and size of the spiral itself, which requires more focus and fine control over the kinematic and dynamic aspects of drawing. In the case of the sentence writing task, Figure 5 shows that the most important pixels tend to be clustered around bends with high curvature. Again, this likely indicates that areas with higher differentiation potential are related to increased demand in terms of the kinematic and dynamic aspects of handwriting. This is an interesting observation showing that a CNN without any knowledge about the evolution of drawing/handwriting over time (as it is given only the final handwritten product) is able to identify the areas in handwritten images that require increased muscular control and focus. This observation could be consistent with the findings presented in Vásquez-Correa et al. (2019), where the transitions from non-moving to moving and from moving to non-moving states were shown to be highly informative. Additionally, this observation supports the importance of handcrafted features and poses an interesting research question of whether deep neural networks, when trained with adequately large and heterogeneous datasets, could provide more insights for the development of new features or whether the present knowledge about baseline handwriting features could be used for the development of novel deep neural networks specialized for automated feature extraction from handwriting/drawing.

### 5.1. Study Limitations

This work has several limitations. First, we need to be aware of the restricted statistical strength of any inferences regarding the population of patients with PD given the relatively limited sample size. In addition, although the clinical information is not complete for all of the datasets, it is evident that the PD cohort contains patients with different levels of PD progression; for example, based on the UPDRS III, the CO subjects are at a more severe stage than the CZ subjects. On the other hand, by fusing them together, we were able to train models that could support the diagnosis of PD in both severe and early stages.

Another limitation is associated with the effect of medication. Since we did not have information about LED for all PD subjects, we could not control for this effect in the statistical modeling. According to Zham et al. (2019), levodopa has a positive effect, especially on the performance in simple graphomotor tasks, such as the Archimedean spiral drawing task in our case. Nevertheless, the authors reported that no such benefit was observed in the sentence writing task, which imposes higher memory and cognitive loads. Therefore, we assume that controlling for the effect of medication in our analyses could further improve the performance of the models based on the spiral drawing task.

Next, although we performed unit unification and resampling on the signals so that they all had the same sampling frequency, the different recording conditions (e.g., paper vs. the display version of the tablets) could still have had some impact on the results.

In addition, various machine learning models should be trained and compared in future studies to obtain more information about the classification performance of the proposed features and to obtain the most robust models for PDYS identification. Finally, the relationship between the classification performance of the trained models and the feature space complexity as well as the cross-validation setup should be investigated to evaluate and confirm the robustness of the proposed methodology.

In summary, considering its limitations, this study should be viewed as a pilot study that is exploratory in nature, and its results should be confirmed by subsequent research studies.

## 6. Conclusion

We investigated several aspects of handwriting evaluation for the detection of PDYS. First, we compared the utilization of handcrafted features with the utilization of features extracted by a CNN. We found that the two approaches are competitive, especially for the spiral drawing task, which is independent of language. Handcrafted features (especially kinematic features) proved to be the better choice for the sentence writing task in multilingual scenarios. This is expected since CNN-based features are extracted only from offline handwriting samples, from which temporal information is not available. In addition, the orthography of a sentence is strongly affected by the language of the writer. Second, we analyzed the effect of multilinguality on the training and performance of classification models. Here, in contrast to our initial hypothesis, model validation performed on sentences written in a different language than the ones used for training did not result in performance degradation. In fact, the prediction accuracy improved in the case of the US and HU datasets. Finally, we compared the sentence writing task and the spiral drawing task. Here, the sentence writing task showed higher discrimination potential, even in multilingual scenarios.

Although there are several limitations, to the best of our knowledge, this is the first study to compare the classification performance of conventional handcrafted features designed by domain experts and features extracted automatically by a pretrained CNN from a multilingual dataset collected from patients suffering from PD. It also provides an objective evaluation of PDYS detection using two different and very promising approaches and analyzes several aspects of handwriting that are frequently neglected in the literature. Based on the results, we can conclude that both types of features have great potential to be used to describe various aspects of drawing/handwriting in both language-independent and language-dependent scenarios. In summary, our work can be perceived as establishing some initial baseline results for further research toward the introduction of new prediction models utilizing handcrafted features as well as CNN-based features that could provide more robustness and confidence in the identification of HD in patients with PD.

## Data Availability Statement

The raw data supporting the conclusions of this article will be made available by the authors, without undue reservation.

## Ethics Statement

The studies involving human participants were reviewed and approved by Research Ethics Committee of Masaryk University, Zerotinovo Namesti, 617/9, 601 77 Brno, Czech Republic. The patients/participants provided their written informed consent to participate in this study.

## Author Contributions

IR, TK, SR, JO-A, MF-Z, ZS, JMe, PD, and ZG: conceptualization. ZG, PD, JMe, MG, JMu, and VZ: research about the current state of knowledge. IR, LB, TK, SR, JO-A, RC, MF-Z, JMe, and ZG: database acquisition, development, and processing. ZG, PD, JMe, MG, JMu, and VZ: feature extraction, machine learning, and experiments. All authors contributed to the article and approved the submitted version.

## Funding

This work was supported by the European Union's Horizon 2020 research and innovation program under the Marie Skłodowska-Curie grant agreement no. 734718 (CoBeN), by the Czech Ministry of Health under grant no. NU20-04-00294, by the Slovak Research and Development Agency under contract no. APVV-16-0211, by the Scientific Grant Agency of the Ministry of Education, Science, Research and Sport of the Slovak Republic and the Slovak Academy of Sciences under contract VEGA 1/0327/20, and by the Spanish grant Ministerio de ciencia e innovación PID2020-113242RB-I00.

## Conflict of Interest

The authors declare that the research was conducted in the absence of any commercial or financial relationships that could be construed as a potential conflict of interest.

## Publisher's Note

All claims expressed in this article are solely those of the authors and do not necessarily represent those of their affiliated organizations, or those of the publisher, the editors and the reviewers. Any product that may be evaluated in this article, or claim that may be made by its manufacturer, is not guaranteed or endorsed by the publisher.
